# c-Myc co-ordinates mRNA cap methylation and ribosomal RNA production

**DOI:** 10.1042/BCJ20160930

**Published:** 2017-01-20

**Authors:** Sianadh Dunn, Olivia Lombardi, Victoria H. Cowling

**Affiliations:** Centre for Gene Regulation and Expression, School of Life Sciences, University of Dundee, Dundee DD1 5EH, U.K.

**Keywords:** eukaryotic gene expression, mRNA cap, RNA, RNA pol I

## Abstract

The mRNA cap is a structure added to RNA pol II transcripts in eukaryotes, which recruits factors involved in RNA processing, nuclear export and translation initiation. RNA guanine-7 methyltransferase (RNMT)–RNA-activating miniprotein (RAM), the mRNA cap methyltransferase complex, completes the basic functional mRNA cap structure, cap 0, by methylating the cap guanosine. Here, we report that RNMT–RAM co-ordinates mRNA processing with ribosome production. Suppression of RNMT–RAM reduces synthesis of the 45S ribosomal RNA (rRNA) precursor. RNMT–RAM is required for c-Myc expression, a major regulator of RNA pol I, which synthesises 45S rRNA. Constitutive expression of c-Myc restores rRNA synthesis when RNMT–RAM is suppressed, indicating that RNMT–RAM controls rRNA production predominantly by controlling c-Myc expression. We report that RNMT–RAM is recruited to the ribosomal DNA locus, which may contribute to rRNA synthesis in certain contexts.

## Introduction

In eukaryotes, gene expression is dependent on the mRNA cap being added to RNA pol II transcripts [1–3]. The cap structure protects transcripts from nucleases and recruits factors that mediate RNA processing, export and translation initiation [4,5]. Transcripts are synthesised with a triphosphate at the 5′ end to which the basic mRNA cap structure, cap 0, is added by the sequential action of three enzymic activities [4,5]. A triphosphatase removes the terminal phosphate and a guanylyltransferase adds guanosine monophosphate to create the cap intermediate, G(5′)ppp(5′)N (N = first transcribed nucleotide). An N-7 RNA methyltransferase catalyses guanosine cap methylation to create the cap 0 structure, m7G(5′)ppp(5′)N. Although the cap can be further methylated on the first transcribed nucleotides, the cap 0 structure is sufficient to recruit cap-binding factors, including CBC (cap-binding complex) and eIF4E (eukaryotic initiation factor 4E), which promote splicing, nuclear export and translation initiation [4,5].

The enzymes that catalyse mRNA cap synthesis are configured in a species-specific manner [6]. In mammals, the triphosphatase and guanylyltransferase are contained in one protein, CE/RNGTT (capping enzyme/RNA guanylyltransferase and 5′-triphosphatase). The methyltransferase, RNMT (RNA guanine-7 methyltransferase), catalyses mRNA cap methylation. RNMT has a cofactor, RAM (RNA-activating miniprotein), which stabilises several regions of RNMT resulting in optimal positioning of key amino acids in the active site [7,8]. RAM also contains an RNA-binding domain that is required for efficient recruitment of transcripts to RNMT [9]. In cancer cell lines, RNMT and RAM expression is co-dependent and they are only found in a complex [7]. Conversely, in embryonic stem cells, RNMT and RAM expression is uncoupled and RAM acts as a signalling molecule [10]. Repression of RAM during the neural differentiation of embryonic stem cells contributes to the remodelling of the gene expression landscape.

Here we demonstrate that RNMT–RAM controls ribosomal RNA (rRNA) production and present the mechanism involved.

## Materials and methods

### Cell culture and treatment

HeLa cells were cultured in DMEM/10% FBS at 37°C and 5% CO_2_. Cells (5 × 10^5^) were transfected with 50–100 nM siRNA (Dharamacon siGenome range; non-targeting, RNMT or RAM) using Lipofectamine RNAiMax (Thermo Fisher Scientific). Cells were infected with INI-based expression plasmids by retroviral transduction and selected using 0.5 mg/ml G418.

### Western blot analysis

Lysis buffer [10 mM Tris (pH 7.05), 50 mM NaCl, 30 mM Na pyrophosphate, 50 mM NaF, 5 µM ZnCl_2_, 10% glycerol, 0.5% Triton X-100 (TX-100), 1 mM EGTA, 1 mM EDTA and 1 mM DTT] was used to extract cellular protein 24–48 h post-siRNA transfection. Western blots were performed to detect RNMT and RAM (own sheep polyclonal antibodies), c-Myc (rabbit polyclonal, Cell Signalling Technology), GAPDH (mouse polyclonal, Abcam), TAFID (goat polyclonal, Santa Cruz Biotechnology), TAF1B (rabbit polyclonal, developed in house), RNA Pol subunit RPA194 (mouse polyclonal, Santa Cruz Biotechnology) and RNA Pol subunit RPA135 (goat polyclonal, Santa Cruz Biotechnology).

### Labelling of cellular RNA with [5,6-^3^H]-uridine or 5-ethynyl uridine

For the labelling of nascent rRNA with [5,6-^3^H]-uridine, cells were incubated with pre-warmed media containing 2.5 µCi/ml [5,6-^3^H]-uridine for 30 min. Cells were washed with cold PBS and RNA was extracted using an RNeasy kit (Qiagen) or TRIzol (Thermo Fisher Scientific). RNA (2 μg) was resolved by denaturing electrophoresis, transferred to Hybond-NX membrane and analysed by autoradiography. Quantification of [5,6-^3^H]-uridine signal in 45S pre-rRNA was determined by Storm phospho-imager and analysed using the AIDA imager analyser software. [5,6-^3^H]-uridine incorporation into total RNA was quantified by scintillation counting using equal amounts (150–300 ng) of RNA. For chase experiments, cells were labelled as above, washed three times in pre-warmed medium and then incubated in pre-warmed media for 30 min, 1 and 2 h. RNA was extracted and analysed as above.

The labelling of nascent rRNA with 5-ethynyl uridine (EU) was performed using the Click-IT RNA Imaging kit (Invitrogen). EU incorporation into RNA was visualised using a Zeiss LSM 700 microscope and quantified by PerkinElmer Volocity software. When used, cells were incubated with 100 ng/ml actinomycin D 30 min prior to EU labelling.

### Immunofluorescence

All incubations were performed in 0.2% BSA/PBS at room temperature unless stated otherwise. Following labelling of RNA by EU, cells were permeabilised in 1% TX-100/PBS for 10 min, blocked with 10% donkey serum for 30 min, incubated with 0.3 ng/ml RNMT antibody for 1 h and incubated with 4 mg/ml Alexa Fluor 488-conjugated donkey anti-sheep antibody (Invitrogen) for 45 min. Cells were counterstained with 1 mg/ml DAPI and visualised by fluorescence microscopy (Zeiss LSM 700). Antibody staining was quantified by PerkinElmer Velocity software.

### Chromatin immunoprecipitation

HeLa cells (10^6^) were transfected with (i) 4 µg of pcDNA4 or (ii) 2 µg of pcDNA4-FLAG-RAM and 2 µg of pcDNA4 HA-RNMT using lipofectamine. Chromatin immunoprecipitations (ChIPs) were performed using the Millipore ChIP kit. A 15 µl aliquot of anti-HA or anti-FLAG antibody-conjugated agarose (Sigma) was used for immunoprecipitation. DNA was purified by phenol:chloroform extraction and precipitated using sodium acetate, using standard protocols. DNA was dissolved in 50 µl of water and 2 µl was used per real-time PCR. ChIP signal was determined relative to the input and control immunoprecipitation signal was subtracted.

### Real-time PCR

Real-time PCR was performed using Quanta Bioscience SYBR Green FastMix for iQ. The ChIP primers used were derived from refs [11–13] H1, forward 5′-GGCGGTTTGAGTGAGACGAGA-3′ and reverse 5′-ACGTGCGCTCACCGAGAGCAG-3′; H4, forward 5′-CGACGACCCATTCGAACGTCT-3′ and reverse 5′-CTCTCCGGAATCGAACCCTGA-3′; H13, forward 5′-ACCTGGCGCTAAACCATTCGT-3′ and reverse 5′-GGACAAACCCTTGTGTCGAGG-3′ and H27, forward 5′-CCTTCCACGAGATGAGAAGCG-3′ and reverse 5′-CTCGACCTCCCGAAATCGTACA-3′; GAPDH (−1407/gene body), forward 5′-CACCCTGGTCTGAGGTTAAATATAG-3′ and reverse 5′-GTGGGAGCACAGGTAAGT-3′.

### Statistical analysis

Statistical significance was assessed using the two-tailed *t*-test using GraphPad Prism 5.0.

## Results

### RNMT–RAM controls rRNA synthesis

**Figure 1.**
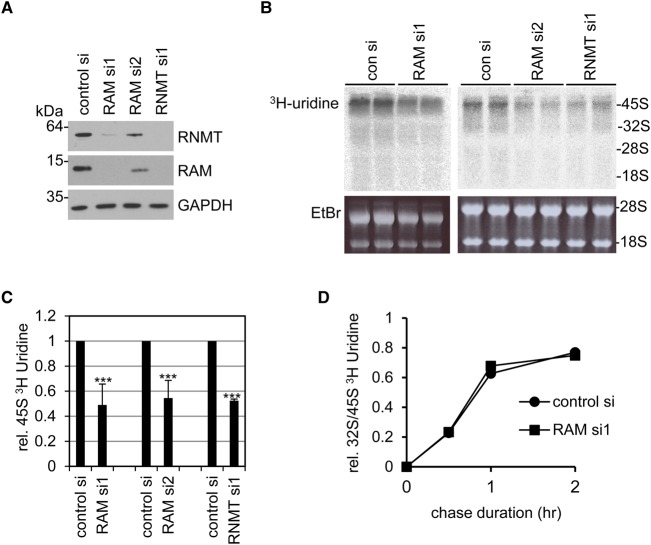
Expression of the mRNA cap methyltransferase complex, RNMT–RAM, is required for 45S rRNA production. HeLa cells were transfected into two independent RAM siRNAs, an RNMT siRNA and a non-targeting control siRNA, for 48 h. (**A**) Expression of RNMT, RAM and GAPDH was analysed by western blot. (**B**) Cells were labelled with [5,6-^3^H]-uridine for 30 min. RNA (2 µg) was resolved by electrophoresis and analysed by autoradiography. Representative autoradiograph is presented, with ethidium bromide-stained gels indicating equivalent loading and migration of 18S and 28S rRNA. (**C**) [5,6-^3^H]-uridine incorporation into 45S RNA in cells transfected with two independent RAM siRNAs, and an RNMT siRNA was determined relative to cells transfected with control siRNA. The average result and standard deviation for four independent experiments (RAM 1 and RAM 2 siRNA) or two independent experiments (RNMT siRNA) are given. Statistical significance was assessed using a two-tailed *t*-test, and a value of *P* ≤ 0.001 is depicted by ***. (**D**) HeLa cells were transfected with a RAM siRNA or a non-targeting control siRNA. After 48 h, cells were pulse-labelled with [5,6-^3^H]-uridine for 30 min and chased for the times indicated. RNA (2 µg) was analysed as above. The ratio of [5,6-^3^H]-uridine incorporation into 45S and 32S rRNA is given. The result is representative of two independent experiments.

To investigate the relationship between the mRNA cap methyltransferase, RNMT–RAM, and rRNA production, HeLa cells were transfected with two independent RAM siRNAs, an RNMT siRNA or a non-targeting control siRNA. Forty-eight hours after transfection of the RNMT and RAM siRNAs, RNMT and RAM expression was reduced (Figure 1A). As observed previously, inhibition of either subunit of the RNMT–RAM complex in HeLa cells resulted in loss of the other [7,9]. rRNA production was initially investigated by incubating cells in [5,6-^3^H]-uridine, which becomes converted into [5,6-^3^H] UTP in the cell and incorporated into nascent RNA. In cells transfected with control siRNA, the nascent 45S rRNA precursor was resolved by gel electrophoresis as a labelled band (Figure 1B). Uridine incorporation into the 45S RNA was reduced following transfection of RAM or RNMT siRNAs (Figure 1B,C). Processing of rRNA was investigated by quantifying the processing of the 45S precursor into the 32S processing intermediate, over a time course following [5,6-^3^H]-uridine labelling. rRNA processing was equivalent in the control and RAM siRNA-transfected cells (Figure 1D).

**Figure 2.**
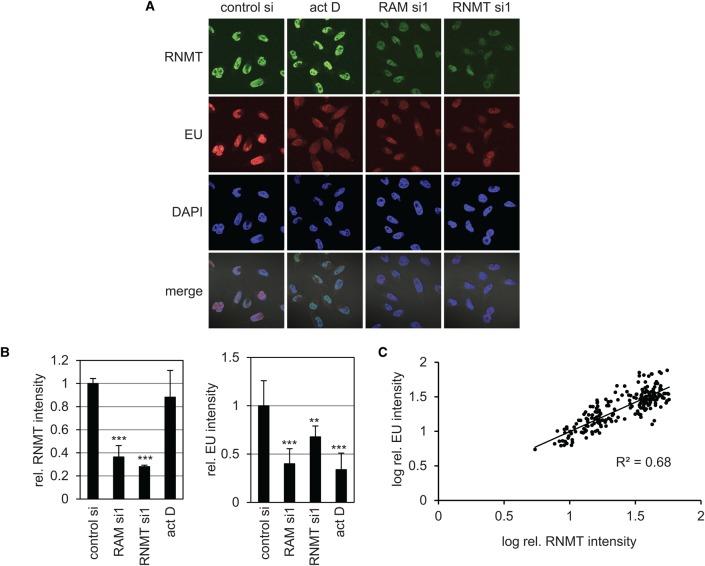
Expression of RNMT–RAM is required for rRNA expression. HeLa cells transfected with RAM, RNMT or a non-targeting control siRNA were labelled with EU for 10 min and analysed using fluorescence microscopy. When used, cells were treated with actinomycin D (Act D) for 30 min prior to EU labelling. RNMT levels were analysed by immunofluorescence microscopy. DAPI staining was used to detect nuclei. (**A**) Representative images from fluorescence microscopy. (**B**) Quantification of cellular RNMT and EU intensity relative to cells transfected with non-targeting siRNA alone. Mean value and standard deviation for 10 images, each containing over 10 cells. Statistical significance was assessed using a two-tailed *t*-test. **P ≤ 0.01 and ***P ≤ 0.001. (**C**) Linear regression analysis of RNMT and EU intensities in cells transfected with RAM, RNMT or non-targeting siRNA. Each point represents an individual cell.

To investigate rRNA synthesis using an independent methodology, cells were incubated with EU, which becomes incorporated into cellular RNA. Incorporated EU can be visualised by a ‘click-reaction’ that links a fluorescent dye to the modified nucleotide (Figure 2A). Although EU will incorporate into all cellular transcripts, since rRNA constitutes 80% cellular RNA, changes in EU signal are a good approximation of changes in rRNA synthesis. EU incorporation was reduced by transfection of RNMT or RAM siRNA (Figure 2A). As a control, EU incorporation was also reduced by the transcription inhibitor actinomycin D (Figure 2A). Quantitation of multiple experiments revealed that transfection of RNMT and RAM siRNA significantly inhibited EU incorporation (Figure 2B). At a single cell level, RNMT protein level (determined by immunofluorescence) and RNA synthesis (determined by EU incorporation) exhibited a positive correlation (Figure 2C).

### RNMT–RAM binds to ribosomal DNA

**Figure 3.**
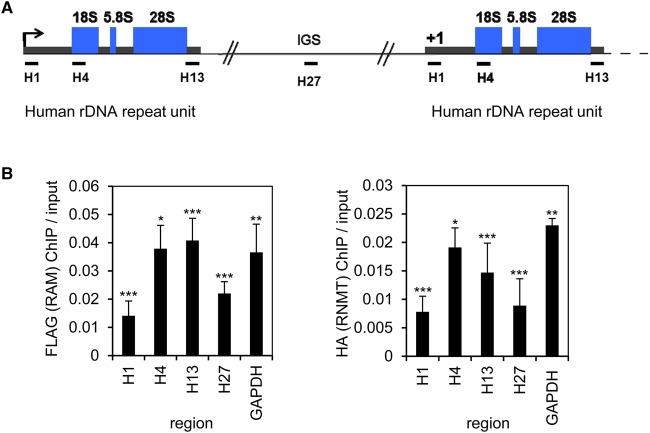
RNMT–RAM is recruited to rDNA. (**A**) Diagram of human rDNA repeat units. (**B**) ChIP was performed on HeLa cells transfected with pcDNA4-HA-RNMT and pcDNA4-FLAG-RAM, or transfected with pcDNA4 as a negative control. ChIPs were performed using anti-HA or anti-FLAG antibodies. DNA was quantified by real-time PCR using the primers indicated beneath the diagram in (**A**). For three independent experiments, the average PCR signal relative to input, with negative control subtracted, is presented. Error bars indicate the standard deviation. Student's two-tailed *t*-test was performed for each PCR from HA-RNMT and FLAG-RAM IP relative to control IP. ***P ≤ 0.001; **P ≤ 0.01; *P ≤ 0.02. IGS is the intergenic spacer region.

rRNA is transcribed from ribosomal DNA (rDNA) repeats (Figure 3A). To investigate whether RNMT–RAM has the potential to influence rRNA transcription directly, ChIP assays were performed to investigate the recruitment of the complex to rDNA. RNMT–RAM binding was analysed throughout the rDNA locus using previously established primers (Figure 3A) [14]. The interaction of endogenous RNMT–RAM with chromatin was difficult to detect, either because the interaction was weak, indirect and/or transient, or because the endogenous antibodies available were not suitable. Therefore, transient transfection was used to simultaneously express HA-RNMT and FLAG-RAM. Cells were treated with formaldehyde to cross-link DNA with protein, and anti-HA and anti-FLAG antibodies were used to immunoprecipitate HA-RNMT and FLAG-RAM. Co-precipitating DNA was purified and specific regions were amplified by PCR. As expected, HA-RNMT and FLAG-RAM were found to bind to the GAPDH gene, proximal to the transcription start site (Figure 3B). HA-RNMT and FLAG-RAM were also found most enriched at the H4 and H13 sites in rDNA (Figure 3B). HA-RNMT and FLAG-RAM were found at lower levels but still significantly bound to the intergenic spacer regions (IGS), 30 kb regions that separate the 13 kb rDNA transcribed regions [15]. RNMT–RAM may potentially be recruited to the paused RNA pol II found at the IGS [16].

### RNMT–RAM controls c-Myc, a regulator of RNA pol I transcription

**Figure 4.**
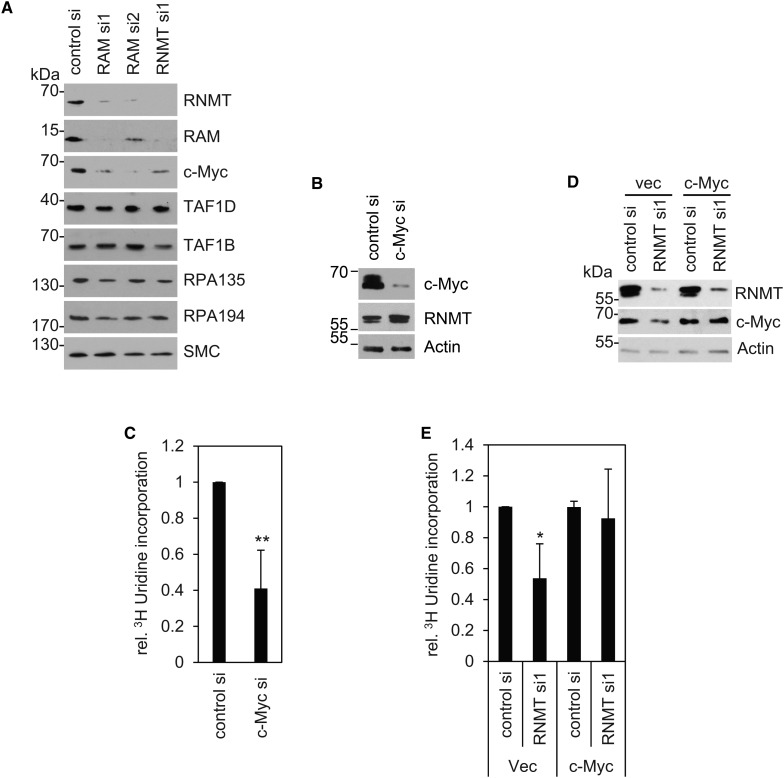
RNMT–RAM regulation of rRNA is dependent on c-Myc. (**A**) HeLa cells were transfected with two independent RAM siRNAs, an RNMT siRNA or a non-targeting control siRNA for 48 h. Expression of RNMT, RAM, c-Myc, TAF1D, TAF1B, RPA135, RPA194 and SMC was analysed by western blot. (**B**) HeLa cells were transfected with control or c-Myc-directed siRNA for 24 h. Western blots were performed to detect c-Myc, RNMT and actin. (**C**) [5,6-^3^H]-uridine incorporation was determined in the same cells (*n* = 5). (**D**) HeLa cells expressing vector control (LXSH) or c-Myc (LXSH c-Myc) were transfected with control or RNMT-directed siRNA for 48 h. Western blots were performed to detect c-Myc, RNMT and actin. (**E**) [5,6-^3^H]-uridine incorporation was determined in the same cells (*n* = 3). For charts, Student's two-tailed *t*-test was performed for uridine incorporation, in cells transfected with gene-specific siRNA relative to control siRNA, **P ≤ 0.01; *P ≤ 0.05. Error bars represent the standard deviation.

Since the major function of RNMT–RAM is to control gene expression by methylating mRNA guanosine caps, we investigated whether it controls the expression of RNA pol I or associated transcription factors. RNMT–RAM expression was suppressed in HeLa cells by transfection of RNMT and RAM siRNA, and the expression of RNA pol I proteins, RPA194 and RPA135, and RNA pol I factors, TAF1D and TAF1B, was investigated (Figure 4A). None of these RNA pol I-associated factors was consistently repressed in response to the level of suppression of RNMT and RAM achieved here. However, expression of c-Myc, a regulator of RNA pol I transcription, was inhibited following suppression of RNMT–RAM. c-Myc was suppressed by transfection of c-Myc siRNA (Figure 4B), and this was confirmed to inhibit rRNA synthesis, as measured using [5,6-^3^H]-uridine incorporation (Figure 4C).

To determine the contribution of c-Myc to RNMT–RAM-dependent rRNA synthesis, retroviral infection was used to constitutively express c-Myc in HeLa cells (Figure 4D). Constitutive expression of c-Myc did not increase c-Myc levels, probably because HeLa cells express high levels of the endogenous protein and additional expression is suppressed by autorepression [17]. However, constitutive expression of c-Myc did maintain its expression when RNM–RAM was suppressed (Figure 4D). As observed previously, repression of RNMT–RAM inhibited rRNA synthesis, determined via [5,6-^3^H]-uridine incorporation (Figure 4E). Constitutive expression of c-Myc fully rescued rRNA synthesis when RNMT–RAM was suppressed. Thus, RNMT–RAM controls rRNA synthesis predominantly by controlling c-Myc expression.

## Discussion

Here, we report that the mammalian mRNA cap methyltransferase, RNMT–RAM, and rRNA synthesis are mechanistically linked, co-ordinating mRNA expression with ribosome production. Co-ordination of the different mechanisms involved in gene expression is likely to be beneficial, since it reduces wastage and aberrant gene expression [18].

The mechanism by which RNMT–RAM controls rRNA production involves c-Myc. c-Myc is an oncogene that regulates transcription and mRNA cap formation [19–22]. In addition, c-Myc regulates RNA pol I production [23–25]. Inhibition of RNMT–RAM expression resulted in a reduction in rRNA synthesis, and this was reversed by constitutive expression of c-Myc. Thus, although RNMT–RAM may control rRNA production by several mechanisms, control of c-Myc is critical. c-Myc also regulates RNA pol III, which produces tRNA and 5S rRNA; therefore, the potential exists for RNMT–RAM to control these transcripts via c-Myc [25].

Although the majority of transcripts are likely to be dependent on RNMT–RAM for expression, some transcripts are more sensitive to RNMT–RAM depletion than others. c-Myc may be particularly sensitive to RNMT–RAM levels, because both the c-Myc transcript and protein have a relatively short half-life [26]. Any mechanism that inhibits transcription or translation leads to a rapid loss of c-Myc, which ideally places the protein to co-ordinate the mechanisms that support gene expression, including ribosome and tRNA production [27]. c-Myc may also be particularly dependent on RNMT–RAM levels because of the configuration of the gene. We have little rationale for why genes are differentially dependent on RNMT–RAM for expression, but this may involve affinity of RNMT–RAM for specific transcript sequences and/or the accessibility of RNMT–RAM to transcripts either due to chromatin context or the rate of transcription [10,28]. Genome-wide RNMT–RNA interaction analysis will be required to address the mechanism of specificity.

In the course of this work, we observed RNMT–RAM recruitment to rDNA loci. The recruitment of RNMT–RAM to rDNA was equivalent to the recruitment to GAPDH, an RNA pol II-dependent gene. However, we did not find a function for RNMT–RAM in rRNA synthesis in HeLa cells. RNMT–RAM is not required for RNA pol I-dependent transcription *in vitro*, and RNMT–RAM was not found to bind to RNA pol I factors (not shown). However, since RNMT–RAM is recruited to rDNA, it may assist RNA pol I-dependent transcription under certain conditions. As discussed above, in HeLa cells, under the conditions used here, the major mechanism by which RNMT–RAM controls rRNA transcription is by regulating c-Myc.

## Abbreviations

ChIPs: chromatin immunoprecipitations
DAPI: 4′,6-diamidino-2-phenylindole, dihydrochloride
DTT DL: dithiothreitol
EDTA: ethylenediaminetetraacetic acid
EGTA: ethylene glycol-bis(2-aminoethylether)-*N*,*N*,*N*′,*N*′-tetraacetic acid
EU: 5-ethynyl uridine
FBS: foetal bovine serum
IGS: intergenic spacer regions
RAM: RNA-activating miniprotein
rDNA: ribosomal DNA
RNMT: RNA guanine-7 methyltransferase
rRNA: ribosomal RNA
TX-100: Triton X-100.
